# Supplementary material for: Divergent mutational processes distinguish hypoxic and normoxic tumours
Source: Nat Commun. 2020 Feb 5;11:737. doi: 10.1038/s41467-019-14052-x (PMC7002770; doi:10.1038/s41467-019-14052-x)
Supplement: Supplementary file 2 — Description of Additional Supplementary Files [file 41467_2019_14052_MOESM2_ESM.pdf]

Description of Additional Supplementary Files

File Name: Supplementary Data 1

Description: Data for hypoxia scores, mutational density/summary, driver mutations, mutational signatures and subclonality for 1,188 tumours from 27 cancer types. For driver mutations, 0 represents wildtype, 1 represents an SNV, 2 represents a CNA, 3 represents an SV and 4 represents a compound event (i.e., those with multiple types of alterations). Mutational signature data for single base substitution signatures and insertion and deletion (ID) signatures are provided as the proportion of alterations attributed to the signature.
